# Neuroecology of alcohol risk and reward: Methanol boosts pheromones and courtship success in *Drosophila melanogaster*

**DOI:** 10.1126/sciadv.adi9683

**Published:** 2025-04-02

**Authors:** Ian W. Keesey, Georg Doll, Sudeshna Das Chakraborty, Amelie Baschwitz, Marion Lemoine, Martin Kaltenpoth, Aleš Svatoš, Silke Sachse, Markus Knaden, Bill S. Hansson

**Affiliations:** ^1^ Max Planck Institute for Chemical Ecology, Department of Evolutionary Neuroethology, Hans-Knöll-Straße 8, D-07745 Jena, Germany.; ^2^ Max Planck Institute for Chemical Ecology, Research Group Olfactory Coding, Hans-Knöll-Straße 8, D-07745 Jena, Germany.; ^3^ European Neuroscience Institute (ENI), Neural Computation and Behavior, Grisebachstraße 5, 37077 Göttingen, Germany.; ^4^ Max Planck Institute for Chemical Ecology, Department of Insect Symbiosis, Hans-Knöll-Straße 8, D-07745 Jena, Germany.; ^5^ Max Planck Institute for Chemical Ecology, Mass Spectrometry/Proteomics Research Group, Hans-Knöll-Straße 8, D-07745 Jena, Germany.

## Abstract

Attraction of *Drosophila melanogaster* toward by-products of alcoholic fermentation, especially ethanol, has been extensively studied. Previous research has provided several interpretations of this attraction, including potential drug abuse, or a self-medicating coping strategy after mate rejection. We posit that the ecologically adaptive value of alcohol attraction has not been fully explored. Here, we assert a simple yet vital biological rationale for this alcohol preference. Flies display attraction to fruits rich in alcohol, specifically ethanol and methanol, where contact results in a rapid amplification of fatty acid–derived pheromones that enhance courtship success. We also identify olfactory sensory neurons that detect these alcohols, where we reveal roles in both attraction and aversion, and show that valence is balanced around alcohol concentration. Moreover, we demonstrate that methanol can be deadly, and adult flies must therefore accurately weigh the trade-off between benefits and costs for exposure within their naturally fermented and alcohol-rich environments.

## Teaser

Flies increase pheromones and mating success with alcohol, but methanol is toxic; thus, the brain must balance risk versus reward.

## INTRODUCTION

Mate selection is of paramount importance in all sexually reproducing species, and for many animals, sexual selection drives exaggerated phenotypes. Thus, the coevolution of senders and receivers of sexual signals, such as pheromones, is an ideal ecological context to study adaptation, speciation, and animal communication. In *Drosophila melanogaster*, like many insects, the female presumably selects a male based on signals of quality. Moreover, in these instances, it is equally necessary for the males of the species to win any competition against their rivals, as it is prevalent in the *Drosophila* genus for several male suitors to court a single female (*1*). Therefore, any competitive advantage in pheromone signaling (e.g., content, abundance, or detection) can have large effects on sexual selection and mating success, as shown in previous studies of *D. melanogaster* (*2*–*7*).

Species within the genus *Drosophila* are known to be variable in host choice and microbial associations (*8*–*11*). However, most species of this genus are united by a common behavioral trait, i.e., their preference for vinegar and fermentation odors. Here, given that *D. melanogaster* is a rotten fruit generalist and a citrus specialist (*12*), where some of the primary by-products of fruit fermentation are copious amounts of alcohols, we focused on the ecological and natural history ramifications of this association between alcohol and the fly.

It has been demonstrated repeatedly that *D. melanogaster* adults (*8*, *13*–*16*) and larvae (*17*, *18*) have a strong behavioral attraction toward ethanol (*14*, *16*, *19*–*22*). We repeat many of these same experiments demonstrating, for example, that virgin males are consistently more attracted to ethanol when compared to recently mated males. Moreover, our current findings support all previous data that have been published addressing the neural links between alcohol and reward pathways in the brain, such as those involving neuropeptide F, as well as the changes in physiological state afforded by mate rejection or by alcohol consumption (*16*, *23*–*25*).

However, in our current paper, we provide substantive alternatives to explain the attraction of *D. melanogaster* toward alcohol from an ecological and evolutionary perspective that is aligned with the natural history of these flies. Previously, authors inferred from the behavioral increases of alcohol consumption in sexually rejected virgin males that this means that flies are using ethanol to cope with the negative feelings caused by the trauma of rejection by the female (*16*). These previous studies often purport that flies are turning to ethanol to activate reward centers in the brain as a drug-like euphoria to counteract the sensations of depression or other negative mood disorders (*26*, *27*). This area of research has led to many publications on the effects of ethanol on the brain of the fly, including the identification of some potentially valuable homologs in the human brain, as related to a new understanding and new interventions for human drug abuse, addictive tendencies, and other negative neurological conditions.

In the present paper, we outline an alternative hypothesis to the same alcohol-related behaviors, namely, that *D. melanogaster* flies, which have evolved over millions of years to subsist in microbe-rich, heavily alcoholic substrates, are attracted to ethanol (and methanol) not as a means to cope with the negative psychological effects of mate rejection, but rather that flies are driven toward these alcohols to increase their chances for subsequent mating success. Moreover, we further support this interpretation by showing that contact with methanol, in particular, markedly increases known, vital, courtship-related pheromones and that flies with access to natural sources of methanol (such as fermenting citrus) consequently outcompete other males who do not have access to these alcohols. In addition, we show that too much methanol is bad and, more specifically, that it can be deadly to the flies. Thus, not all alcohol in the environment is inherently good. We also demonstrate that this behavioral risk and reward related to alcohol exposure seems to be regulated by several different but complimentary olfactory receptor pathways that detect methanol, where one pathway detects lower concentrations and appears to drive attraction, while the other circuit detects higher concentrations and dictates aversion.

Therefore, this paper promotes the ethological adaptive value of the observed behavioral attraction of flies toward ethanol and methanol, specifically that exposure increases pheromones that enhance courtship success, an interpretation that more closely aligns with the natural history of *D. melanogaster*, a species that has evolved within its preferred, highly alcoholic host materials.

## RESULTS

### Flywalk behavioral assay

**Fig. 1.**
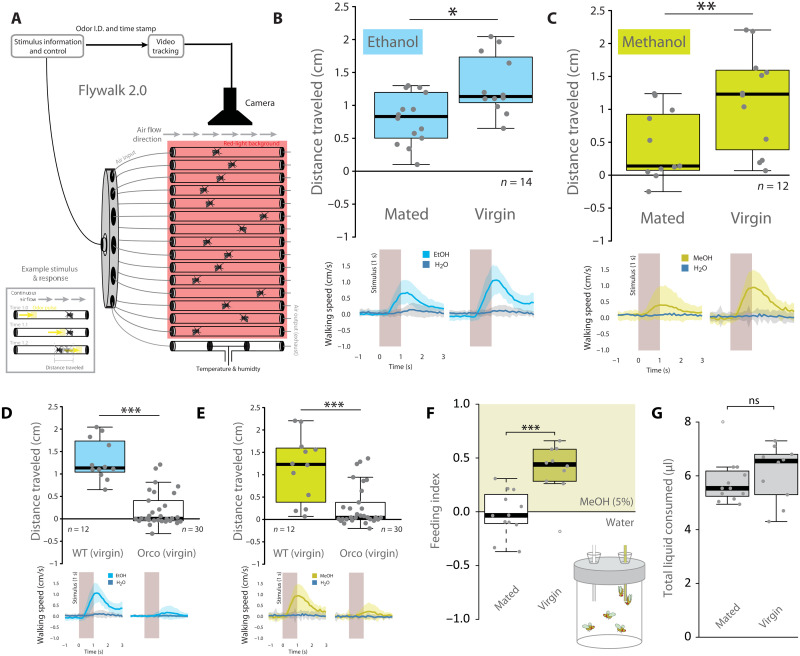
Behavioral attraction of *Drosophila* toward alcohols. (**A**) Flywalk design schematic for simultaneous video recording and analyses of odor-evoked behavioral responses across 15 flies. Inset box: Example stimulus of odor pulse (in yellow) moving down the tube over time, eliciting an insect response (forward, upwind movement, and subsequent distance traveled after pulse exposure). Each of the flies is exposed to identical odorant pulses, well defined in quality, quantity, stimulus direction, and stimulus duration. Communication between the stimulus delivery system and the automated computer tracking system allows calculation of the exact time of the encounter between the odor stimulus and the fly (i.e., the meeting time based on wind speed, stimulus onset, and position of the fly within the glass tube). This calculation enables detailed analysis of the behavior of individual flies before, during, and after odor stimulation. Odor I.D., odor identity. (**B** and **C**) Flywalk upwind attraction for mated and virgin males relative to water (control), for both ethanol (EtOH; in blue) and methanol (MeOH; in yellow). Bottom panels depict walking speed before and after arrival of a 1-s stimulus (gray bar) of either EtOH, MeOH, or water. Flywalk upwind attraction toward (**D**) EtOH or (**E**) MeOH for wild-type (WT) and Orco mutant virgin males. (**F** and **G**) Feeding preference (F) and total liquid consumption (G) of virgin and mated males measured in CAFÉ assay. All panels: Box plots depict medians (black bold lines), quartiles (boxes), and maximum as well as minimum values (whiskers). *P < 0.05, **P < 0.01, ***P < 0.001, two-tailed unpaired *t* test. For calculations in (B) from the bottom panel to the upper panel, we use the equation of distance equals rate multiplied by time (*d* = *r* × *t*). ns, not significant.

In order to examine how flies interact with sources of alcohol, we conducted behavioral studies of attraction (Fig. 1, A to C). In these experiments, we used a behavioral assay known as the Flywalk, a high-throughput paradigm for studying odorant-evoked behavior in walking *Drosophila*. This assay allows the simultaneous computer-assisted tracking of 15 freely walking flies arranged in parallel using small-sized wind tunnels (length, 18 cm; inner diameter, 0.8 cm; constant airflow, 18 cm/s) (Fig. 1A). Each of the flies is exposed to identical odorant pulses, well defined in quality, quantity, stimulus direction, and stimulus duration. Communication between the stimulus delivery system and the automated computer tracking system allows calculation of the exact time of the encounter between the odor stimulus and the fly (i.e., the meeting time based on wind speed, stimulus onset, and the position of the fly within the glass tube). This calculation enables detailed analysis of the behavior of individual flies before, during, and after odor stimulation (*28*, *29*). The Flywalk also has the advantage of keeping single flies housed in entirely separate glass tubes (chemically and heat-cleaned between every trial), which reduces any confounding variables such as social interactions or fly-deposited residues. We collect streaming data in the Flywalk that allow us to determine both the rate (speed) of the flies and the time they are in motion following each stimulation (Fig. 1B, bottom); thus, we are able to quickly calculate the distance that the flies moved as a result of each individual pulse of the test odorant (or control) over the course of the experiment (where we show the average responses from each fly to 40 total pulses spaced randomly over the duration). Flies generally remained motionless or moved randomly during the pre-odor period. In addition, flies did not change their movements upon meeting with pulses of the control, in this case, toward clean air. After a fly encountered a pulse of ethanol (Fig. 1B), a known attractant (*8*, *14*, *16*, *20*), the fly exhibited rapidly increased upwind walking speed (i.e., toward the odor source) (Fig. 1B), where the distance traveled after an odor pulse has been shown to be a good proxy for the relative strength of an odorant’s attraction (*28*, *29*). We found a similar attraction of male flies toward methanol, i.e., a compound that to our knowledge before had not been tested regarding its valence.

The Flywalk offered an ideal design to test these very volatile substances, as it is a largely closed system, with odor sources held in sealed chambers until delivered to the animal. This meant that we could be confident over the duration of each experiment that all flies were always getting the same consistent dose of ethanol and methanol during each exposure. This was particularly important for our cohorts of flies, as we later show that lower concentrations are highly attractive (3 to 5%), while higher concentrations result in aversion (15%).

### Behavioral responses of flies toward ethanol and methanol

As shown previously in the literature (*15*, *16*, *20*, *30*–*34*), male flies were also acutely attracted to sources of ethanol in the Flywalk (Fig. 1B). Furthermore, we confirmed that mating status created variability in this attractive behavior (*16*), namely, that virgin males were more attracted to sources of ethanol than recently mated males (Fig. 1B). In addition, we could show that a similar or even stronger increase in behavioral attraction was present for methanol (Fig. 1C), which has not been previously reported. Next, we demonstrated that this attraction is attributed to olfaction by testing Orco anosmic mutants (*35*), where anosmic flies were no longer attracted to ethanol or methanol in behavioral trials (Fig. 1, D and E). Our result is in agreement with current studies using adults (*31*), but less so with previous research using larvae, which have suggested that ethanol is, perhaps, detected by more than just Orco-mediated means. However, even in these cases, Orco mutant larvae were greatly reduced in their ethanol attraction (*15*). To our knowledge, in studies that have found that *D. melanogaster* attraction is retained while testing Orco mutants, each of these studies has generally used ethanol and standard food medium as the combined stimulus, and has not tested ethanol alone (*15*, *36*). Similarly, we readdressed alcohol feeding preferences across male physiological states to confirm previous results (*16*) and could show that virgin males clearly prefer to consume alcohol, in this case, specifically methanol, when compared to mated males (Fig. 1, F and G), a compound that was not examined in these previous studies that focused on ethanol. Here, all males drank statistically identical volumes of liquids; thus, the preference for methanol cannot be explained by increased total liquid consumption (Fig. 1, F and G).

### Ecologically relevant sources of ethanol and methanol

**Fig. 2.**
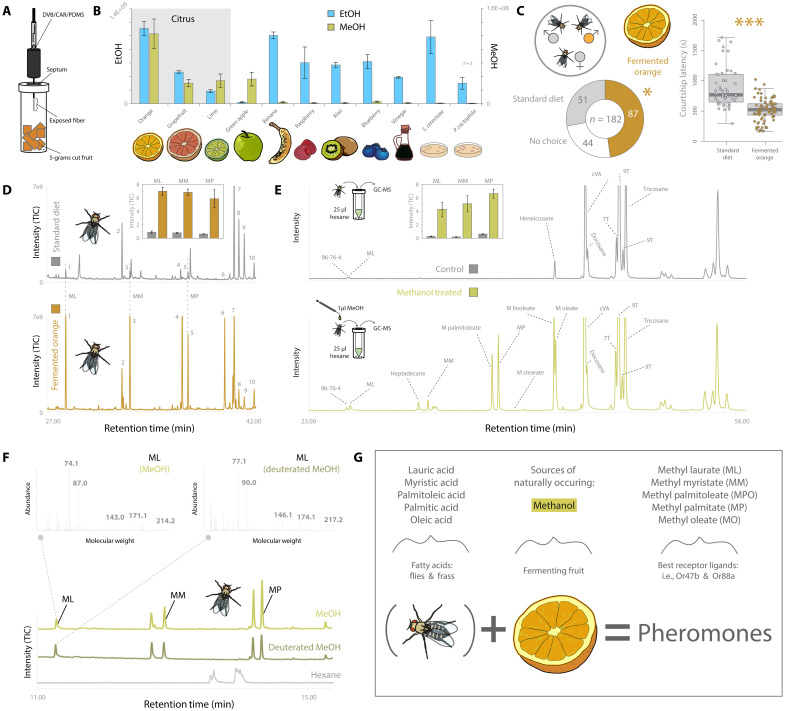
Host chemistry, fly courtship, and fly pheromone association with alcohols. (**A**) Solid-phase microextraction (SPME) technique for odorant collection from host substrates, including highly volatile odors such as ethanol and methanol. (**B**) Quantification of ethanol and methanol content from a variety of natural sources. (**C**) Competitive courtship between two males, one from fermented citrus and the other from standard diet alone (*P* = 0.02). Courtship latency associated with these mating trials (paired *t* test, *P* < 0.001). (**D**) Thermal desorption unit (TDU) analyses of male flies reared on standard diet with only water (top; in gray) or with fermenting orange (bottom; in orange). Inset figure: Changes of main pheromones [including ML (1), MM (3), and MP (5)] following exposure to standard diet with water or orange. TIC, total ion chromatogram. (**E**) Chemical profiles of male flies established using body wash techniques with and without exposure to methanol (bottom and top, respectively). Inset figure: See above. (**F**) Chemical profiles of male flies that were exposed to either methanol or deuterated methanol with shifts in mass spectral peaks after exposure to deuterated methanol. (**G**) Schematic overview of alcohol exposure leading to pheromone increase.

We next sought to ascertain which natural host resources produced the highest levels of alcohol (i.e., ethanol and methanol) during fermentation (Fig. 2, A and B, and fig. S1, A and B). Here, we could show that citrus fruits, which contain high amounts of pectin (*37*–*41*), produced equal or higher amounts of both ethanol and methanol relative to all other tested resources (Fig. 2B). In this context, it is again important to note that *D. melanogaster* has already been shown to strongly prefer citrus as host fruits (*12*), including studies concerning the ancestral, citrus-like host plants from its African origins (*42*).

### Increased courtship success and pheromone enhancement after alcohol exposure

Next, we examined whether males exposed to fermenting citrus had any advantage when in competition with males from a standard laboratory diet. In this experiment, males exposed to fermented orange significantly outperformed their competition in copulation success (Fig. 2C), where in those trials where copulation occurred, 63% were from orange, while 37% were from standard diet (*P* = 0.02). Moreover, flies that were exposed to fermenting citrus were also observed to have significantly lower copulation latency (mean = 517 s) again compared to laboratory diet flies (mean = 889 s) (*t* test, *P* < 0.001). Our findings consistently support the notion that exposure to ecologically relevant and alcohol-containing natural host substrates results in a courtship and mating advantage.

We then compared the odor profiles of males that had been allowed contact with either citrus fruits (i.e., organic, fermented, and sliced oranges) or standard laboratory diet alone (Fig. 2D and fig. S2A). In the citrus-exposed animals, but not in those fed only laboratory diets, we observed a marked increase in several previously established pheromone components, including fatty acid esters known to be involved in both courtship and aggregation behaviors (*2*, *7*, *43*–*45*). This included increases in pheromones such as methyl laurate (ML), methyl myristate (MM), methyl palmitate (MP), and methyl palmitoleate (Fig. 2D). In order to examine the involvement of alcohols in the augmented pheromone titers, we pursued experiments with body washes of adult males with and without exposure to methanol and ethanol (Fig. 2E and figs. S2 and S3). Increased production of fatty acid ester pheromones was only observed when the flies had contacted these alcohols, with distinctly larger increases in pheromone release or production for methanol as opposed to ethanol. This observation matches previous research showing that pheromone-specific olfactory sensory neurons (OSNs) and the olfactory receptors they express (i.e., Or47b and Or88a) respond more strongly to methyl rather than ethyl esters of these fatty acids (*2*, *43*, *45*). To examine any direct role of alcohol in pheromone biosynthesis, we subsequently used deuterated isotopologues of methanol (i.e., CD_3_OD instead of CH_3_OH), where these solvents differed only in their isotopic composition, i.e., deuterium replaces hydrogen. These results in deuterated methanol have a distinct shift in mass spectrum, which we could observe during gas chromatography–mass spectrometry (GC-MS) analyses. All observed increases in the pheromone profile of the fly following contact with the deuterated methanol resulted in deuterated pheromone compounds (Fig. 2F and fig. S3). Therefore, we assert that contacted methanol directly donated hydrogen atoms (or, more likely, -CH_3_ methyl groups) to form fatty acid methyl ester pheromones from fatty acid precursors on the male fly following exposure to methanol (Fig. 2G). This simple donation from an alcohol of a single methyl group likely explains the majority of the biosynthetic pathway from fatty acid precursor to fatty acid ester (i.e., pheromone) in *D. melanogaster* adults once direct contact is made with methanol. However, putting high-purity synthetic fatty acids into pure alcohols only produced a 1% to 3% yield of the pheromone compounds (fig. S1C). Thus, an additional catalyst from the fly must still be required to complete this biosynthetic pathway in nature. Subsequent chemical analyses with insects that were devoid of microorganisms (i.e., axenic versus conventional flies) showed that the required catalyst does not originate from a yeast or bacterium, but rather from the fly itself (fig. S4). However, the exact biosynthetic pathways for pheromone production or increased pheromone release of ML, MM, and MP as well as the incorporation of methanol hydrogens or methyl groups (-CH_3_) remain elusive. Future research will need to be conducted to unravel the specific biochemical process that incorporates ethanol and methanol to accelerate or increase overall production and release of the courtship pheromones, where we note that the fatty acid precursors and the fat body are primarily found in the adult abdomen (*46*); thus, this is a likely place to start tracing increased biosynthesis of these pheromones after contact with natural sources of alcohol.

### Olfactory detection of alcohols

**Fig. 3.**
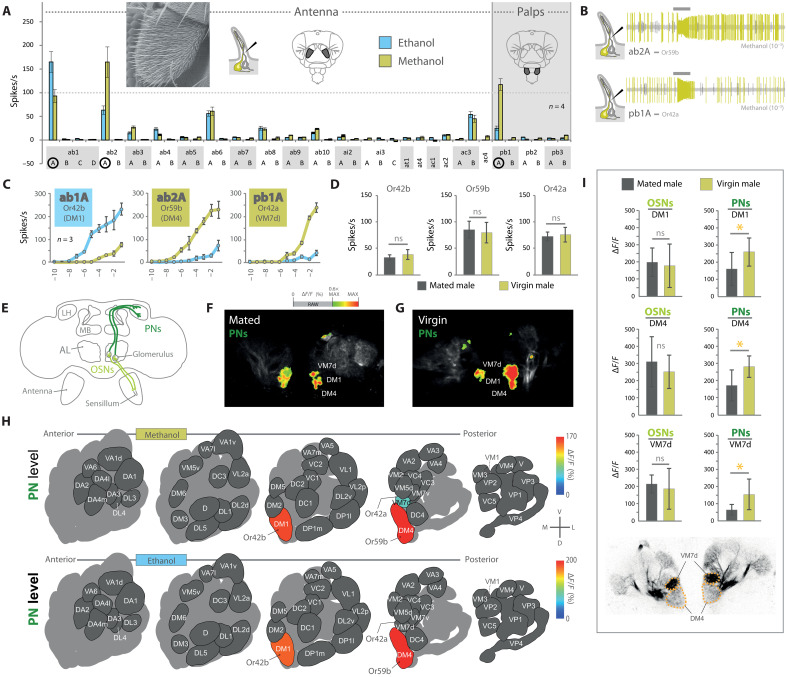
Peripheral and central brain pathways for alcohol detection. (**A**) Entire olfactory screen of all olfactory sensillum types across both the antenna and palps of adult wild-type flies using SSR technique with three sensilla displaying a response of over 100 spikes/s toward alcohols. (**B**) Example traces of methanol responses for antennal basiconic 2 (ab2) and palp basiconic 1 (pb1). (**C**) Dose-response curves toward both alcohols of neurons identified in (A). (**D**) SSR responses of mated and virgin males to methanol (ns, *P* > 0.05, two-tailed unpaired *t* test). (**E**) Diagram of *D. melanogaster* brain, highlighting OSN and PN circuits. (**F** and **G**) Representative odor-evoked calcium responses in PNs from mated and virgin males responding to methanol obtained through two-photon functional imaging (more in fig. S7). (**H**) Schematic AL atlas showing odor-induced calcium responses obtained with two-photon functional imaging toward methanol and ethanol (10^−3^) in olfactory PNs. Data collected from mated males. (**I**) Odor-induced calcium activity toward methanol (10^−3^) as recorded from OSN input (*n* = 5 to 9) and PN input (*n* = 8 to 9) (*P < 0.05, two-tailed unpaired *t* test). Bottom: Gray-scale image represents AL structure with identified glomeruli of interest.

Given the behavioral motivation of *D. melanogaster* for contact with alcohols, we subsequently examined the mechanism by which the fly detects these odors (Fig. 3). Whole antenna electroantennogram (EAG) and maxillary palp recordings showed that both structures react to ethanol and methanol (fig. S5). Genetic mutants for the olfactory co-receptor Orco no longer detected either alcohol, nor produced behavioral attraction, suggesting again that odorant receptors (ORs) are necessary for alcohol detection (Fig. 1, D and E, and fig. S5, A and B). We additionally confirmed the OR-dependent detection using mutants deficient in both ionotropic-mediated co-receptors (i.e., Ir8a and Ir25a), which did not show any deficiency in alcohol detection (fig. S5C). Next, we pursued single sensillum recordings (SSRs) to ascertain which of the *D. melanogaster* ORs were specifically involved (Fig. 3, A to C). OSNs expressing three different ORs were found to be clearly activated by ethanol and methanol, including displaying dose-dependent responses (Fig. 3, A to C, and fig. S5H). While additional receptors showed low to moderate responses to ethanol and methanol (Fig. 3A), such as sensillum ab6A (expressing Or13a) and sensillum ac3B (expressing Or35a), both of these receptors have also been shown to be widely tuned and shown to respond to many odorants, especially those that have an active -OH group attached (*47*, *48*). The largest SSR responses to the alcohols were observed in OSN type ab1A (which expresses Or42b) with a stronger affinity to ethanol and in ab2A (Or59b) and pb1A (Or42a) with stronger responses toward methanol. These results differ slightly from previous papers addressing these receptors (*48*–*50*). This may be because ethanol has sometimes been used to dilute other compounds that are subsequently delivered in odor stimulation screens (*2*), where ethanol or methanol was used as a control solvent, and would be allowed to evaporate before animal testing. It is also likely that due to the high volatility of ethanol and methanol, these odorants were sometimes lost to the environment before delivery over the animal in previous EAG or SSR screens. Here, while previous studies outline ethyl acetate (for Or42b) and methyl acetate (for Or59b) as the strongest ligands for these receptors (*48*–*53*), these compounds (i.e., ethyl and methyl acetate) are also intimately tied to the presence of naturally occurring ethanol and methanol, respectively, including the biosynthetic process of fruit host fermentation. Moreover, in our natural sampling of fermenting resources using GC-MS headspace (Fig. 2B), we did not find ethyl acetate or methyl acetate without the presence of ethanol and methanol. In our electrophysiology trials, we also noted a synergy between ethanol exposure and at1A response to cis-vaccenyl acetate (cVA) (which has been demonstrated previously) (*34*, *54*, *55*), as well as a possible synergy between at4A responses toward methanol exposure and ML (fig. S5, D and E). Neither alcohol directly activated these pheromone-detecting, trichoid-associated OSNs when presented alone. In addition, at the OSN level, we did not observe any variation in SSR response toward methanol based on mating status (mated versus nonmated males) (Fig. 3D).

### Neurobiology of alcohol detection pathways in the brain

Moving from the periphery into the brain, using two-photon functional imaging of calcium dynamics in both OSNs and projection neurons (PNs), we found that two glomeruli within the antennal lobe (AL) were strongly activated by methanol and ethanol after stimulation of OSNs on the antenna (Fig. 3E and figs. S5, F and G, and S7, B and C). This included DM1 (innervated by OSN ab1A expressing Or42b) and DM4 (ab2A and Or59b). In correspondence with the SSR responses of the two innervating OSN types, the two glomeruli were preferentially tuned toward either ethanol (DM1) or methanol (DM4), respectively (Fig. 3, A to C, and fig. S5, F and G). After stimulation of OSNs on the maxillary palps, a third glomerulus, VM7d (pb1A and Or42a), was also found to be activated in the AL. The affinity of this palp OSN was clearly higher toward methanol than ethanol, although with lower overall sensitivity than that observed in DM4 from the antenna (Fig. 3, A, C, and E). All three glomeruli that detected ethanol and methanol were close proximity neighbors within the AL, suggesting a similar evolutionary origin of these primary detection centers for alcohols (Fig. 3H) (*56*).

In order to revisit the role of mating status, which produced variation in behavioral attraction toward sources of alcohol (Fig. 1) (*16*), we next examined at what neural level we could observe this variance within the brain. Here, mated and virgin males showed no difference in sensitivity at the level of OSNs, at least toward methanol, neither in SSR nor in calcium imaging of OSN responses in the AL (Fig. 3, D to F and I, and fig. S5, F, G, and I). Moreover, virgin males displayed significantly higher sensitivity toward methanol when we imaged calcium dynamics in PNs, which represent the output elements of the AL (Fig. 3, F to I, and fig. S5K). However, virgin males did show an increased OSN response of the DM1 glomerulus toward ethanol when compared to those that had recently mated (fig. S5I). For methanol, we thus conclude that a potential neuronal rationale to the state-dependent increase in attraction toward alcohol appeared in neural connections toward higher brain structures [i.e., mushroom body (MB) and lateral horn (LH)] and after processing within the AL. In addition, this state-dependent change was not observed at the sensory periphery (i.e., antenna and palps) nor across the input side of the AL, for methanol, specifically. Furthermore, we note that this mating status–dependent increase in PN activity was specific to this alcohol circuit and did not appear within neural pathways related to navigation toward food resources (fig. S5J). We thus provide evidence for a three-part input and output processing pathway for alcohol detection in the AL, as well as the role of PNs but not OSNs in modulating these state-dependent behaviors, at least for methanol.

**Fig. 4.**
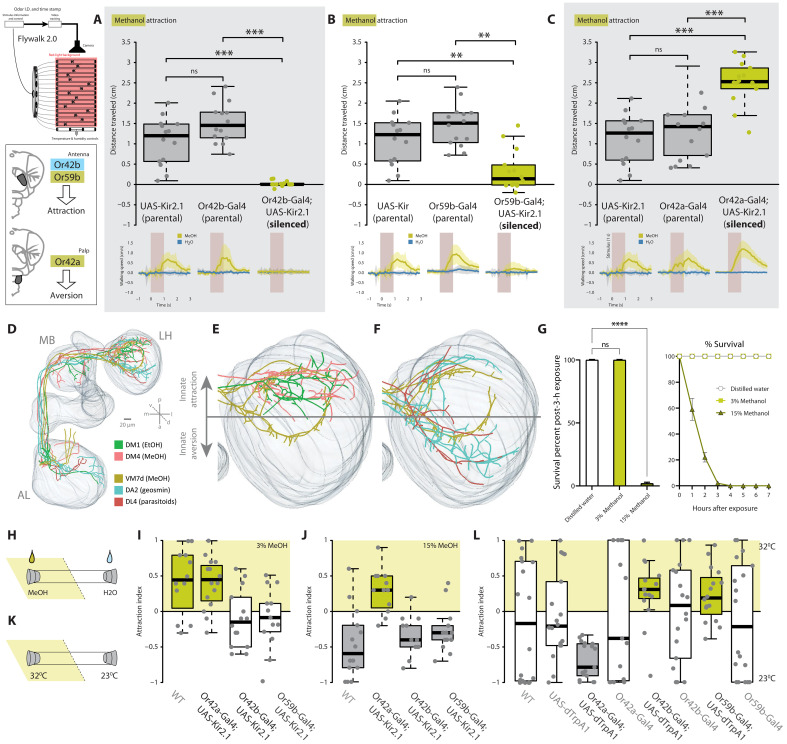
Neural circuitry guiding behavioral valence for alcohol. Flywalk behavior and upwind attraction for wild-type and single-neuron silenced virgin males to methanol. Bottom panels depict walking speed before and after arrival of a 1-s stimulus (gray bar) of methanol or water. UAS-Kir2.1 parental lines are replotted for each comparison to loss-of-function lines of (**A**) Or42b (ab1A and DM1), (**B**) Or59b (ab2A and DM4), and (**C**) Or42a (pb1A and VM7d). [(A) to (C)] *P < 0.05, **P < 0.01, ***P < 0.001, analysis of variance (ANOVA) with Bonferroni correction for selected pairs to correct for repeated comparison with UAS-Kir2.1 parental line. (**D**) Complete neural circuit reconstruction of PNs for DM1, DM4, and VM7d (i.e., all alcohol-detecting circuits) in comparison to two known aversive circuits emerging from DA2 (geosmin detection) and DL4 (parasitoid detection). (**E**) Neural circuit reconstruction of PNs extending into the LH for DM1, DM4, and VM7d (i.e., all alcohol-detecting circuits). The two circuits related to attraction (DM1 and DM4) target the upper portions of the LH that is related to innate attraction according to previous studies, while the PNs from our potentially aversive circuit innervate the lower part of the LH (VM7d). (**F**) Overlay of our potentially aversive circuit (VM7d) with neural circuits from PNs for two well-studied labeled lines for aversion (DA2 and DL4). (**G**) Survivorship after exposure to two ecologically relevant methanol concentrations. (**H**) Schematic of tube assay to test for concentration-dependent attractiveness. (**I** and **J**) Behavioral responses of wild-type, genetic controls, and single-circuit silenced flies toward low (3% MeOH) (I) and high concentrations (15% MeOH) (J) of methanol. (**K**) Schematic of tube assay to thermally activate single circuits in the absence of odors. (**L**) Responses of wild-type, genetic controls, and single-circuit activated flies to thermal activation [yellow boxes, significant attraction (*P* < 0.05); gray boxes, significant aversion (*P* < 0.05); white boxes, not significant; one-sample *t* test].

Many of the previously characterized dedicated circuits for olfactory behavior use only a single neural pathway (*12*, *30*, *57*); hence, we next wanted to explore why these flies have three separate pathways for alcohol detection. We revisited attraction paradigms using transgenic flies, where we tested flies with individually silenced OSNs expressing one of the three main alcohol-detecting receptors (Fig. 4, A to C). While each silenced OR on the antenna (i.e., Or42b and Or59b) caused a pronounced loss of attraction compared to parental controls, the third silenced OR (Or42a), located on the palps, resulted in a significant increase in attraction. This suggested that two circuits relate to attraction toward alcohols, while the third potentially mediates aversion toward methanol. Contrary to our expectations, where we predicted one receptor would be overwhelmingly responsible for attraction, we observed that silencing either Or42b or Or59b resulted in an almost complete loss of attraction in the Flywalk (Fig. 4, A and B). Future research will need to address possible overlapping signaling cascades and neural innervation related to these two receptors at both the OSN and PN levels.

Using single PN labeling through photoactivatable green fluorescent protein (GFP) and subsequent neuronal reconstructions (*58*), in combination with already published materials (*59*–*61*), we next assessed the innervation patterns from the AL toward the MB and LH for all three alcohol-related circuits (Fig. 4, D to F, and fig. S6). Two alcohol pathways displayed a high degree of overlap (i.e., DM1 and DM4, both innervating from the antenna, and coding for alcohol attraction) with major branches in the MB and overlapping innervation areas in the LH. However, the third (emerging from VM7d; innervating from the palp and coding for methanol aversion) displayed only minor branches within the MB and a clearly separate innervation area within the LH (Fig. 4E). Moreover, the innervation pattern of this pathway (VM7d) matched well with other known aversive circuits in the MB and LH (Fig. 4F and fig. S6, B, G, and H). This difference in innervation pattern was not due to VM7d originating from the palps, as opposed to the antenna (fig. S6E). Hence, PN labeling and reconstruction of our three circuits further support the findings of our behavioral assessment, where Or42a-activated PNs display typical characteristics of an aversive circuit, while Or42b- and Or59b-activated PNs match characteristics of attractive pathways. Thus, in summary, behavioral and neural data are in agreement that of these three receptor circuits, two appear to be attractive, while a third (from the palps) appears to be aversive. It should be noted that this study did not explore neural computations between these pathways or neuroactivity manipulations; therefore, behavioral differences in physiological state (i.e., mated and virgin males) and the behavioral differences toward concentrations of alcohols should for now be considered separate issues to address in the future.

### Risks and rewards for methanol exposure in flies

Why would *D. melanogaster* maintain pathways for both attraction and aversion toward the same odorant? In this case, we identified that both ethanol and methanol are toxic at higher concentrations, with methanol more so than ethanol, and thus, contact bears an inherent risk for the flies (Fig. 4G). We tested ecologically relevant concentrations of methanol, similar to what flies might experience in nature. While all flies survived exposure to 3% methanol (identical survivorship to water controls), we observed a 100% mortality for flies exposed to 15% methanol after only a few hours (Fig. 4G).

We further hypothesized that alcohols might therefore be attractive at low concentrations, but necessarily more aversive at higher concentrations. Again, using single-receptor loss-of-function mutants, we tested attraction at both low (3%) and high (15%) ecologically relevant concentrations of methanol (Fig. 4, H to J) (*62*, *63*). In these experiments, we observed that two ORs were necessary for attraction to low methanol levels (Or42b and Or59b), while aversion to high methanol content was dictated by a single receptor (Or42a). Furthermore, we artificially activated these three circuits independently and without odor stimulation using thermogenetics, and could again surmise that while two receptors drive attraction, the third receptor (Or42a) was sufficient to drive aversion even when this OR was activated by heat alone (Fig. 4, K and L).

## DISCUSSION

Male *D. melanogaster* actively prefer and choose to contact sources of higher relative methanol content, such as citrus fruits. This motivation for methanol is stronger in virgin males than in those that have recently mated. Contact with methanol-rich citrus fruit produces an escalation in the production of aggregation and courtship pheromones, which subsequently leads directly to increased male mating success (Fig. 2C), a behavioral result supported by previous literature on these pheromones (*2*, *7*, *43*, *64*). While methanol itself is a highly volatile and fleeting odor, contact with this alcohol contributes to increased production of a more environmentally stable pheromone signal (i.e., ML). This thereby can advertise the ability of a male to successfully find and use optimal stages of host decay or signal the ability of the male to withstand high concentrations of otherwise toxic alcohols (*65*). Moreover, the increased attraction to alcohol by virgin males can give a direct advantage in subsequent courtship, where we show that males have enhanced courtship success after contacting food sources with high methanol content (Fig. 2C). We present this as a simple yet vital biological rationale for alcohol seeking by these insects, as opposed to depression or substance abuse interpretations of this behavior (*16*, *26*, *27*, *34*). It has also been well documented across many model organisms that individuals that produce more pheromone are preferred over rivals in mate competition (*2*–*5*, *66*–*68*). We also identify three sensory neural pathways that control attraction and aversion to alcohols, namely, Or59b and Or42b, each found on the antenna, as well as Or42a, which is located on the palps.

Previous literature has repeatedly documented the roles that fatty acid pheromones play in *D. melanogaster* courtship, mate acceptance, and mating success, as well as social or aggregation behaviors (*2*–*4*, *7*, *43*–*45*, *69*). This includes specific studies that show how pheromone abundance on a male fly decreases subsequent copulation latency and increases copulation success and, moreover, how these methylated pheromones specifically activate only two OSNs found within the antennal trichoid sensillum type at4 (*2*, *43*). These at4 OSNs express Or47b and Or88a and detect ML, MM, MP, and methyl palmitoleate. Previous studies also show that knocking down the pheromone-specific receptor Or47b results in a marked reduction in male mating success (~80% less) versus wild-type control rivals in mate competition. (*2*, *7*, *64*, *69*). It has thus been conclusively shown that these pheromones, as well as their associated olfactory receptors, are directly related to the courtship advantages observed between males when competing for the same female (*2*–*4*). Moreover, the scientific literature broadly shows that animals emitting more pheromone often win significantly more mate competitions against rivals. Similar pheromone trends have been found in other laboratory models, such as nematodes (*68*) and house mice (*66*), as well as other insects, such as wasps (*67*) and bark beetles (*70*), where individuals or aggregations that produce more pheromone have a mating and reproductive advantage and are more preferred in any associated courtship competition. Other pheromones, such as cuticular hydrocarbons (CHCs), have also been shown to affect social behaviors, including courtship in *D. melanogaster* (*5*, *6*). Here, we did not identify any changes to CHCs or cVA (produced in male accessory gland) after contact with methanol (Fig. 2, D and E).

The biosynthetic pathways underpinning the production of fatty acid methyl ester pheromones in *Drosophila* have remained a mystery since their discovery a decade ago (*2*, *43*). In laboratory reared flies on standard diets, these pheromones are produced in barely detectable amounts. Our current paper proposes a simple biosynthetic pathway that quickly and efficiently converts abundant fatty acids (e.g., lauric acid) found on the fly into pheromone and courtship enhancing fatty acid methyl esters (e.g., ML) (Fig. 2, D to G). Contact with ecologically relevant amounts of alcohols, such as that found in fermenting orange, results in a pheromone increase in the order of 100× to 1000× more when comparing flies with alcohol contact to those from standard diet alone. Thus, these pheromone- and courtship-related compounds were something that was essentially hidden from the scientific community, perhaps largely due to colonies of flies being reared on standard laboratory food medium and not being reared on natural, ecologically relevant (and strongly behaviorally preferred) fermenting fruit substrates. Moreover, this fatty acid biosynthesis is well supported by literature from lepidopteran pheromone production (*71*, *72*). These studies of other insects have shown the effects of diet and host materials on adult pheromone production and courtship success (*73*–*76*), but this biosynthesis was not previously found concerning the well-known tight associations of alcohol and *D. melanogaster*.

It is unusual in *Drosophila* neurobiology to have two receptors tuned to the same odorant, such as we observe in Or42a and Or59b. However, these results are similar to studies of ecologically relevant concentrations of carbon dioxide, where two neural pathways for the same key ligand have been characterized and are weighed against each other in the brain in order to control differences in behavioral valence (*30*). Cross-talk in the brain has also been shown to occur between information regarding different types of odorants (*54*, *55*, *77*). This communication in primary neural processing, e.g., in the AL via local interneurons, may play a larger role in physiological state-dependent coding (*78*), as well as in determining feeding and oviposition preferences within and across the *Drosophila* genus toward the same chemical cues (*79*, *80*). For example, this may occur in divergent preferences for different stages of host fermentation across the genus, where degree of fermentation can result in large changes in both carbon dioxide and alcohol content (*81**–**83*). In addition, recent studies across 20 *Drosophila* species have shown that both of the attractive circuits for alcohol (i.e., Or42b and Or59b) are highly conserved within the *Sophophora* subgenus of *Drosophila* in regard to both amino acid sequence and olfactory function (*81*). This further suggests a strong evolutionary conserved role for alcohol detection across dozens of closely related species. While this evolutionary conservation remains to be shown for the aversive alcohol pathway (Or42a) found on the palps (which was not examined in these previous studies), future research should address the cross-talk between these neural circuits that control the relative valence of alcohols in different *Drosophila* species, especially in those species that do not strongly prefer fermented fruit resources, such as the fresh fruit breeder *D. suzukii* and the flower breeding *D. elegans*.

In summary, we show that *D. melanogaster* are strongly attracted to methanol and that this contact results in a strong pheromone enhancement and associated courtship benefits. We also show that methanol contact is inherently dangerous, as it is acutely toxic at moderately elevated concentrations (Fig. 4G). Separate positive and negative methanol-coding pathways in the brain provide the neural substrate necessary for behavioral decisions and risk assessments by the fly. Thus, adult *D. melanogaster* must carefully examine the benefits versus costs for alcohol exposure, where the rewards might sometimes outweigh the risks, especially for virgin or rejected males that are seeking any advantage to compete successfully for a mate.

## MATERIALS AND METHODS

### Fly stocks and insect rearing

Transgenic fly lines were obtained from the Bloomington *Drosophila* Stock Center (BDSC; http://flystocks.bio.indiana.edu/), and diets for rearing are included within the Supplementary Materials. Unless otherwise noted, all fly stocks were maintained on standard diet (normal food) or yeast food (i.e., axenic assays) at 25°C with a 12-hour light/dark cycle in 70% humidity. Stock population density and size were controlled using 20 to 25 females per vial. Stocks were maintained according to previous publications (*84*), and for all behavioral experiments, we used 2- to 7-day-old male flies. For mating status experiments, we collected males as virgins and then later divided them into three cohorts. We gave one group of males access to intact, virgin females and allowed them to mate and complete copulation within 1 hour before the onset of behavioral experiments (e.g., mated). A second group was not ever exposed to any females (i.e., virgin). Thus, we controlled for age of the males, with the only differences occurring within the 1-hour window time before the onset of the behavioral experiments. Hence, we have focused solely on the behavior and neurological changes between mated and virgin males. For imaging, we used *Orco-Gal4* (from A. Fiala), *GH146-Gal4* (from L. Vosshall’s laboratory), and *20XUAS-IVS-GCaMP6f* (VK00005) (BDSC; 52869). Three- to seven-day-old males with different mating state, either virgin or mated, were imaged. Further fly stocks used: *D. melanogaster*, wild-type Canton S (BDSC, #64349), END1-2, UAS-C3PA, MZ699-GAL4 (*85*), Ir8a/Ir25a double-null mutants (from Y. Grosjean and R. Benton) (*69*, *86*), UAS-Kir2.1 (*87*), Or59b-Gal4, Or42b-Gal4, Or42a-Gal4 (*88*), and UAS-dTrpA1 (*89*).

### Flywalk attraction assay

Behavioral experiments were performed in the Flywalk paradigm as previously described (*28*, *90*), except with male flies starved for only 1 to 3 hours before the start of the experiments. In short, 15 individual flies were placed in 15 glass tubes (inner diameter, 0.8 cm). Glass tubes were aligned in parallel, and flies were continuously monitored by an overhead camera (Sony EVI, Sony Corporation, Japan) under red-light conditions (λ > 630 nm) (Fig. 1A). During the experiment, flies were continuously exposed to a humidified airflow of 20 cm/s (70% relative humidity at 20°C). We presented flies repeatedly with pulses of different olfactory stimuli at an interstimulus interval of 90 s. Stimuli were added to the continuous airstream and thus traveled through the glass tubes at a constant speed. Odor stimulation was performed with a multicomponent stimulus device described elsewhere (*28*, *90*). In summary, 100 μl of an odor was prepared in 200 μl of polymerase chain reaction tubes without cap, where these tubes were placed in odor containers made of polyetheretherketone thermoplastic. These odor containers were tightly sealed (to prevent any evaporation of high-volatility odorants, such as ethanol and methanol) and connected to the stimulus device via ball-stop check valves. These valves only allow unidirectional airflow through the odor-saturated headspace. Odor stimulation was achieved by switching an airflow otherwise passing through an empty vial (compensatory airflow) to the odor-containing vial. Odor pulses were 500 ms in duration, again at an interstimulus interval of 90 s. Different stimuli were presented in pseudorandomized order (random block design) to avoid odor-sequence artifacts. A control stimulation was always included in the randomized block, which consisted of only the humidified water. Please see the included diagrams for more complete descriptions of these behavioral assays.

### CAFÉ feeding assay

All tested flies were 2 to 7 days old, which included only males, and flies were starved beforehand for 18 to 20 hours with constant access to water. Flies were then cooled for 5 min at −20°C to assist in their transfer (without CO_2_) to plastic vial arenas (Fig. 1D). Basic feeding solutions consisted of water with 5% sucrose, as well as 5% MeOH (treatment) or without (control). The capillary feeder (CAFÉ) assays used glass micropipettes with liquid medium that were filled by capillary action and then inserted through pipette tips into the container holding the adult flies (*44*). Each assay had both control and treatment options for feeding. The volume consumed from each capillary was measured after a set duration. The feeding index was calculated as (treatment − control)/total volume consumed.

### Trap assay with fruit choice

We collected virgin males and sorted them immediately after pupation. All males were then kept in cohorts of less than 20 with access to food. Mated males were generated by collecting virgin males after a 1 hour of exposure to twice as many virgin females to ensure successful mating had occurred. One hour before the experiments, flies were again sorted on CO_2_ pads and counted into groups of 30 individuals, and at this time they began starvation with access to only water for 2 hours. Both orange and banana were selected from an organic market, and cut and weighed into 5-g replicates. Fruit replicates were individually fermented at 29° to 32°C for 48 hours in previously autoclaved glass containers with minimal airflow. Any fruit that developed visible mold was not further used. After fermentation, the fruit was transferred into plastic containers with colored paper cone (lobster trap) entrances and set up within 50 cm–by–50 cm–by–50 cm white cages (*84*, *91*). Males were allowed to choose between the fruit for 24 hours (12-hour light:12-hour dark, 70% humidity, 25°C) before removal, collection, and counts of their fruit choice. A total of 10 replicates were conducted per trial, with 30 males per experiment.

### Odor collections, SSR, GC-MS, and TDU–GC-MS

All synthetic odorants that were tested in this publication were acquired from commercial sources (Sigma-Aldrich, www.sigmaaldrich.com; Bedoukian, www.bedoukian.com) and were of the highest purity available. Stimuli preparation and delivery for behavioral experiments followed previously established procedures, and collection of volatile and nonvolatile compounds was carried out according to standard procedures (*44*, *45*, *83*). GC-MS (HP5 and HP-Innowax; liquid samples) and thermal desorption unit (TDU)–GC-MS analyses (HP5 and HP-Innowax; single fly, solid samples) were performed on all odor collections and insect body washes as described previously (*43*). The National Institute of Standards and Technology (NIST) mass spectral library identifications were confirmed with chemical standards where available, and the internal standard bromodecane was used for quantification and statistical comparisons between analyzed pheromone samples. SSR experiments were conducted as described previously (*43*, *45*, *81*). Adult flies were immobilized in pipette tips, and the third antennal segment or the palps were placed in a stable position onto a glass coverslip. Sensilla were localized under a microscope (BX51WI, Olympus) at 100× magnification, and the extracellular signals originating from the OSNs were measured by inserting a tungsten wire electrode into the base of a single sensillum. The reference electrode was inserted into the compound eye. Signals were amplified (10×, Syntech Universal AC/DC Probe, Syntech), sampled (10,667.0 samples/s), and filtered (30 to 3000 Hz, with 50/60-Hz suppression) via USB-IDAC4 computer connection (Syntech). Action potentials were extracted using Auto Spike 32 software (Syntech, v3.7). Neuron activities were recorded for 10 s, with a stimulation duration of 0.5 s. Two milliliters of the diluted odors was added to glass bottle (50 ml; Duran Group, Mainz, Germany), where this bottle was equipped with two sealed openings for the input and output of the airflow. This greatly decreased any evaporation of high volatility odorants, such as ethanol and methanol, used during electrophysiology experiments. Responses from individual OSNs were calculated as the increase (or decrease) in the action potential frequency (spikes per second) relative to the prestimulus spike frequency.

Concentrations of alcohols were calculated from freshly fermented samples. Five grams of each organic fruit type was collected into sealed glass vials and allowed to ferment with minimal airflow for 2 days. Solid-phase microextraction (SPME) using triple-action fibers (DVB/CAR/PDMS) provided collection of all volatile odorants from each sample, replicated three times. Fibers were cleaned between collections using manufacturer recommended heating protocols under clean helium streams. Peak area for each alcohol was calculated and averaged across all three replicates. Alcohol content from both yeasts was measured from 5-ml liquid cultures, using identical glass vials, SPME fiber, and volumes as solid host materials. Methanol and ethanol are shown at different scales (Fig. 2B), with ethanol 10× times higher.

### Deuterated solvents

Methanol and ethanol of the highest possible purity were used wherever possible [>99.5% purity, reinst, CP43.1 (MeOH), 5054.2 (EtOH); Carl Roth GmbH]. For deuterated solvents, we used previously published guidelines (*92*) to identify shifted mass spectral peaks between methanol- and deuterated methanol–treated adult male flies (D4, 100Atom%D, CAS: 811-98-3; Carl Roth GmbH).

### Two-photon calcium imaging (OSNs, PNs)

We dissected male flies for calcium imaging according to our standard protocol (*55*, *93*). In brief, flies were briefly immobilized on ice and then mounted onto a custom-made stage. Protemp II composite (3M ESPE) was used to fix the head. We bent the anterior part of the fly’s head with a fine gold wire, and a small plastic plate having a round window was placed on top. We sealed the head with that plate using two-component silicone (Kwik Sil), leaving the center part open to make a cut. The cuticle between the eyes and the ocelli was cut under saline solution (130 mM NaCl, 5 mM KCl, 2 mM MgCl_2_, 2 mM CaCl_2_, 36 mM saccharose, 5 mM Hepes, 1 M NaOH, pH 7.3). The cuticle was either bent forward and fixed to the silicon or removed. After cleaning the fatty tissues and trachea, we were able to visualize the AL.

We used a two-photon laser scanning microscope (2PCLSM, Zeiss LSM 710 meta NLO) equipped with an infrared Chameleon Ultra diode-pumped laser (Coherent, Santa Clara, CA) and a 40× water immersion objective lens (W Plan-Apochromat 40×/1.0 DIC M27). Among the odorants, methanol (99.5% from Sigma-Aldrich) and ethanol (99.5% from Sigma-Aldrich) were diluted in Milli-Q water to concentrations of 10^−3^, 10^−4^, 10^−5^ and 10^−6^. The odor dilutions were made freshly every day. Ethyl hexanoate was diluted in mineral oil to a concentration of 10^−4^. Two milliliters of odor with different dilutions was pipetted into a glass bottle (50 ml, Duran Group, Mainz, Germany), which had two sealed openings for the in-and-out of the airflow. For odor delivery, we used a computer-controlled odor delivery system (as described previously) (*77*). Odor stimuli were injected into this airstream after 2 s of the beginning of the recording for a duration of 2 s. A series of 40 images was acquired at a resolution of 256 × 256 pixels with a recording frequency of 4 Hz (i.e., 10 s) in total. Each animal has been imaged at three to four different focal planes to monitor calcium responses in different glomeruli from the entire AL. Each odor was measured only once in each animal and the odor stimulation sequence was delivered from low concentration to high concentration for each experiment. The interstimulus interval was at least 60 s to avoid any effects of adaptation or habituation.

We analyzed the obtained imaging data with custom-written IDL 6.4 software (ITT Visual Information Solutions). Manual movement correction and bleach corrections were followed by the calculation of relative fluorescence changes (Δ*F*/*F*) from the background. Individual glomeruli were identified with the help of the digital in vivo three-dimensional (3D) AL atlas (*57*) according to previous publications (*55*, *77*). The Δ*F*/*F* of all 40 frames was imported to Excel and further analyzed. The responses from frames 10 to 18 were averaged for the glomerulus of interest for all treatments and defined as the odor response. Unpaired, two-tailed *t* test was used for all statistical analyses of the imaging data.

### Neural 3D reconstruction, tracing, and mapping

For in vivo photoactivation experiments, 1- to 6-day-old flies (genotype: END1-2, UAS-C3PA; MZ699- GAL4) (*85*) were dissected, and the tracts of the salivary glands were cut to prevent movement. Photoactivation was accomplished at a two-photon microscope (same as used for the functional imaging experiments) via continuous illumination with 760 nm for 15 to 25 min. After a 5-min break to permit full diffusion of the photoconverted molecules, 925-nm z-stacks of the whole brain were acquired and subsequently used for neuronal 3D reconstruction. Z-stacks were performed with the Chameleon Laser 925 nm and had a resolution of 1024 or 512 square pixels. The maximum step size for immunopreparations or single-neuron projections was 1 μm, and for AL reconstructions 2 μm. For all 3D reconstructions, we used the segmentation software AMIRA 4.1.1 & 5.3.3 (FEI Visualization Sciences Group, Burlington, MA). Neurons of different individuals were embedded into the reference brain using a label-field registration as previously described (*94*). Briefly, segmented labels of brain neuropils (AL, MB, and LH) were registered onto a reference brain image using affine registration followed by elastic warping. In a second step, the calculated transformation matrix was applied to the respective neuron morphology that was then aligned to the reference brain image (*85*).

Reconstructions of single neurons were also compared to previously published online datasets, including those obtained from Virtual Fly Brain (VFB; https://v2.virtualflybrain.org/). VFB uses an ontological model of *D. melanogaster* anatomy written in OWL2 and based on the *Drosophila* literature. This contains detailed information about gross neuroanatomy, neuron classes, as well as the relationships between them. Underlying each neuroanatomy query is a query of this ontology in OWL-DL. Queries of phenotype and expression first use the large volume of expression and phenotype data available from FlyBase (https://flybase.org/) and then annotate using the *Drosophila* anatomy ontology. Each expression or phenotype query starts with a query of the anatomy ontology for terms appropriate to the chosen region. The output of this query is then used as input for a query of the FlyBase database for expression or phenotype annotated using these terms. Images in the online viewer are delivered as a series of tiles covering only the visible area in the browser window. The tiles are produced from a compound 3D Woolz object (https://github.com/ma-tech/Woolz), representing the overall structure and individual color painted domains. Here, we use the neural tracings of the following PNs: DM1 (VFB_00101219), DM4 (VFB_00101234), Vm7d (VFB_00101137), DA2 (VFB_00101260), DL4 (VFB_00101235), and VC2 (VFB_00101165).

### Tube assays (with odorants or temperature gradients)

Male flies were collected shortly after emergence, as described previously for virgins. We kept all flies at 22° to 23°C in 70% humidity for 24 hours within the behavioral chambers, prior to onset of their use in experiments. Single adults were placed into tubes described from Flywalk assays, but without dynamic headspace. Each end of the tube was sealed with medical-grade cotton, and an aliquot of 50 μl of water (control) or 50 μl of alcohol diluted in water (treatment) was added to opposite sides of the tube. We allowed flies to acclimate for 10 min without disturbance, and then we recorded their resting position within the tube after an additional 5 min of observation. We used the fly position (in centimeters) from the control side to generate an attraction index, with higher numbers indicating proximity to the treatment. For heat sensitive loss-of-function or heat activation lines, we used a similar assay consisting of the same glass tubes. However, instead of odors, one side of the glass tube was heated to 30° to 35°C (treatment), while the other side remained at ambient temperature (20° to 24°C). A Bosch PTD 1 laser thermometer (Robert Bosch Power Tools GmbH; Stuttgart, Germany) recorded the temperatures of both sides of the glass tubes at the onset of each experiment, where three glass tubes and fly replicates were usually run in parallel.

### Toxicity and alcohol exposure

Two hundred microliters of distilled water (solvent) or methanol (3 or 15% diluted in water) was pipetted in the cap of a 15-ml Falcon tube. A porous foam plug in the cap circumvented direct contact of the flies with the solution, while a headspace in the Falcon tube could be established. Ten flies (8 days old, mixed sexes, not starved) were exposed in the Falcon tube to the headspace, and their survival rate was quantified on an hourly basis. Bar plots depict average survival rates (%) of 20 replicates per test condition after 3 hours of exposure (Fig. 4G). Error bars depict SD. Statistical significance was assessed using the Kruskal-Wallis test with Dunn’s post hoc test for multiple comparisons. 

### Courtship

All adults were collected as virgins and kept in separate vials until use in this assay. In the competition mating assays (two males, one female), both males were marked by ultraviolet (UV) fluorescent powder of different colors. Fluorescent powder was purchased from Amazon (https://amazon.com; powder visible only at UV 365-nm black light and water based). This courtship assay was allowed to run for 30 min and observed manually, where mating success was recorded by identifying the color of the successful fly under UV light after copulation with female. If no mating occurred in allotted time, assay was scored as no choice. Before assay, male flies were exposed to either standard diet in rearing vials overnight or standard diet vials plus fermenting orange (fruit was previously fermented for 72 hours in airtight container, and orange material was confirmed to be without any mold at time of use after fermenting).

### Generation of axenic and conventional flies

Axenic flies were generated using modifications toward previously established protocols (*95*), in this case to collect first-instar larvae, and these methods are further described herein. Adult females were mated and then allowed to oviposit their eggs onto apple-juice plates. Before egg transfer, we cleaned tools and the new fly food for rearing with ethanol and with exposure to UV light for 15 min inside a sterile bench for starvation medium (i.e., apple-juice plates), where the eggs were then transferred after the bleaching step. The apple-juice plates where the flies laid eggs were not sterilized before use, but the medium was autoclaved and poured in the clean bench. After oviposition, we rinsed the apple-juice plates with phosphate-buffered saline (PBS) 1×, gently brushed the surface, and then poured the contents into two sieves for egg collection. The collection contents from both sieves were washed three times with PBST (0.1% Triton X), resuspended for 35 s, and then rinsed again with distilled water. Next, in the clean bench, the eggs were washed in 3% bleach solution by transferring over another sieve into a sterilized container and subsequently cleaned again with distilled water. All eggs, including axenic and conventional, were stored separately for 1 day in an incubator at 22°C with 60% humidity until larvae could emerge and be collected. Here again, the axenic fly larvae were kept to food medium that was previously autoclaved and then poured into tubes in the clean bench. We exposed these food vials to UV light and kept them in sterile closed containers until use.

### Statistics and figure preparation

Statistical analyses were conducted using GraphPad InStat 3.1 (https://www.graphpad.com/scientific-software/instat/) and R Project (https://www.r-project.org/), while figures were organized and prepared using R Studio, Microsoft Excel, and Adobe Illustrator CS5. Flywalk data (distance traveled), preference data (attraction index), survivorship data, and CAFÉ assay data (feeding index) were examined using a Mann-Whitney *U* test. Attraction and feeding indices were calculated as (*O* − *C*)/*T*, where *O* is the number of flies observed in the treatment, *C* is the number of flies in the control, and *T* is the total number of flies used in the trial. The courtship assays were assessed with a chi-square test of frequency. Imaging data were assessed using a two-tailed, unpaired *t* test (assuming Gaussian distribution). Error bars for bar graphs represent SD. Boxplots represent the median (bold black line), quartiles (boxes), as well as 1.5 times the interquartile range (whiskers). Filled boxes denote significance from zero. Statistics were performed using GraphPad InStat version 3.1 at α = 0.05 (*), α = 0.01 (**), and α = 0.001 (***) levels.
