# Supplementary material for: Neuroecology of alcohol risk and reward: Methanol boosts pheromones and courtship success in Drosophila melanogaster
Source: Sci Adv. 2025 Apr 2;11(14):eadi9683. doi: 10.1126/sciadv.adi9683 (PMC11963984; doi:10.1126/sciadv.adi9683)
Supplement: Supplementary file 1 — Figs. S1 to S7 Legend for data S1 [file sciadv.adi9683_sm.pdf]

Science Advances
AAAS

Supplementary Materials for

Neuroecology of alcohol risk and reward: Methanol boosts pheromones and courtship success in *Drosophila melanogaster*

Ian W. Keesey *et al.*

Corresponding author: Bill S. Hansson, hansson@ice.mpg.de

Sci. Adv. **11**, eadi9683 (2025)
DOI: 10.1126/sciadv.adi9683

The PDF file includes:

Figs. S1 to S7
Legend for data S1

Other Supplementary Material for this manuscript includes the following:

Data S1

Figure S1

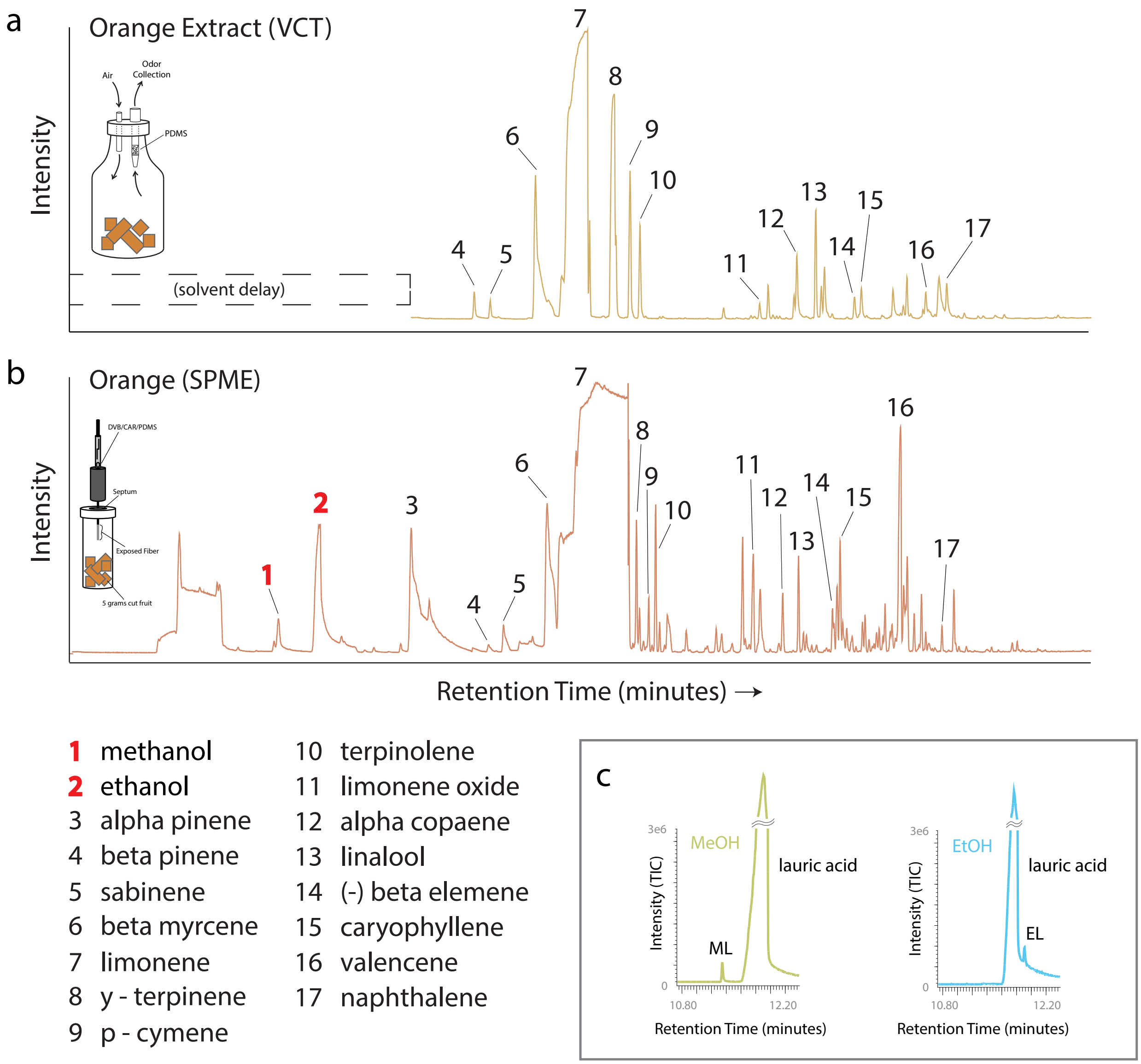

Figure S1. Odor samples from fermenting oranges.
In order to examine the roles of the numerous compounds generated from orange fruit material potentially involved in the increased production of pheromone components after fly exposure to this fermenting fruit, we collected samples in two ways. Shown are the GC-MS total ion chromatograms (TICs) from each collection method using identical host plant materials. (a) The headspace from sliced oranges was collected using volatile collection traps (VCTs; PDMS absorbent), and then eluted using hexane solvent. (b) The headspace of sliced oranges was collected using solid phase micro extraction (SPME), and then analyzed by injection into the same GC-MS. Here we note nearly identical odor identities collected with these two sampling techniques. However, importantly, VCT collections did not contain highly volatile odors when compared to SPME (which are lost during sample collection), and this would include the loss of both alcohols. (c) Putting lauric acid in alcohols only gives 1-3% yield of pheromone (therefore a potential catalyst from fly or microorganism appears to be missing).

Figure S2

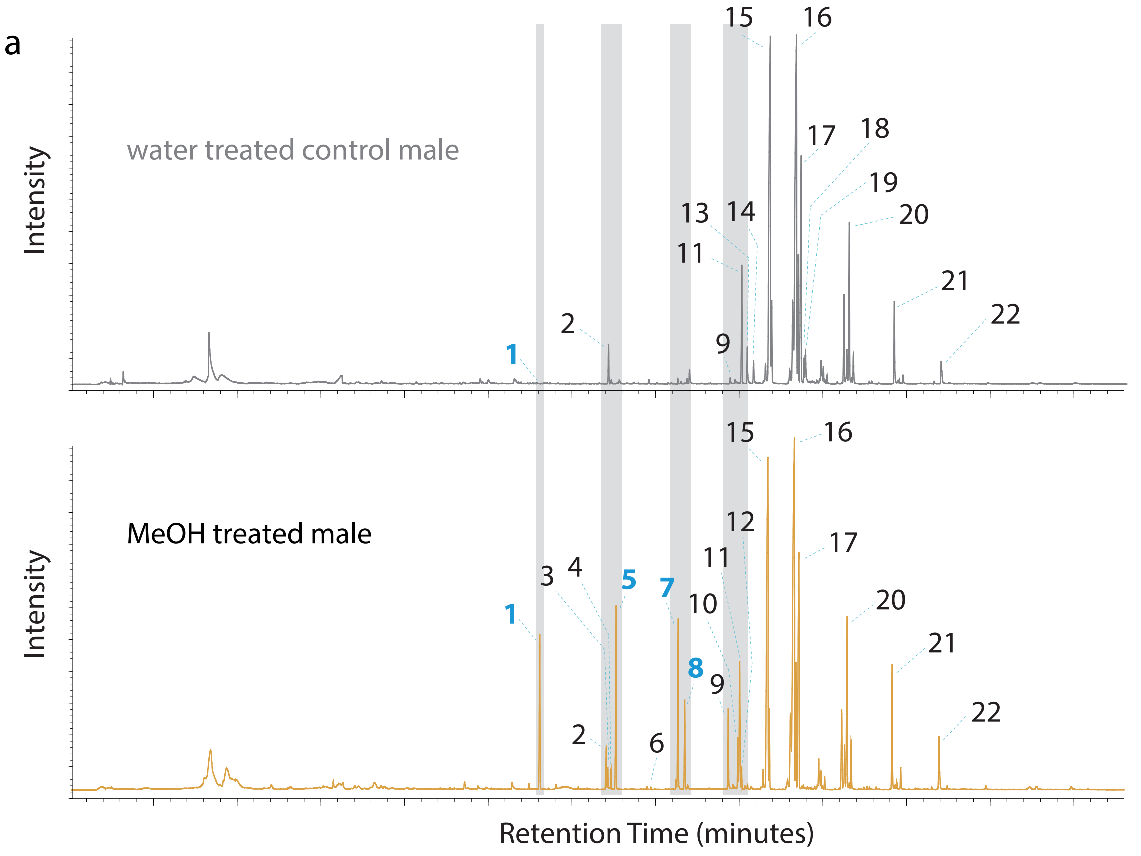

1	Methyl laurate	11	Heneicosane
2	Heptadecane	12	Methyl oleate
3	Methyl myristoleate (A)	13	Palmidrol (A)
4	Methyl myristoleate (B)	14	Palmidrol (B)
5	Methyl myristate	15	cVA
6	Methyl pentadecanoate	16	7-tricosane
7	Methyl palmitoleate	17	Tricosane
8	Methyl palmitate	18	Palmidrol (C)
9	Oleyl alcohol	19	Palmidrol (D)
10	Methyl linoleate	20	7(Z)-Pentacosene
		21	2-methylhexacosane
		22	2-methyloctacosane

increased for MeOH treated flies

common to both control and treatment

higher in control flies

Figure S2. TDU-GC-MS chemical analyses of adult males.
In order to assess the chemical profile of adult males in the absence of solvent injection, we utilized a single, live fly thermal desorption unit (TDU). Here we either pre-treated adults with a 1ul drop of water (top; grey) or a 1ul droplet of 10% methanol (MeOH; diluted in water; bottom, orange). We then thermally desorbed live flies into the GC-MS. Here we noted increases for several, but not all body odors emanating from the insect. For example, we did not observe any changes to cuticular hydrocarbons (CHCs) or for cVA (which is produced by the male accessory glands). However, we again note strong increases for several fatty acid methyl esters, including behaviorally active ML, MM, and MP (peaks 1, 5, and 8).

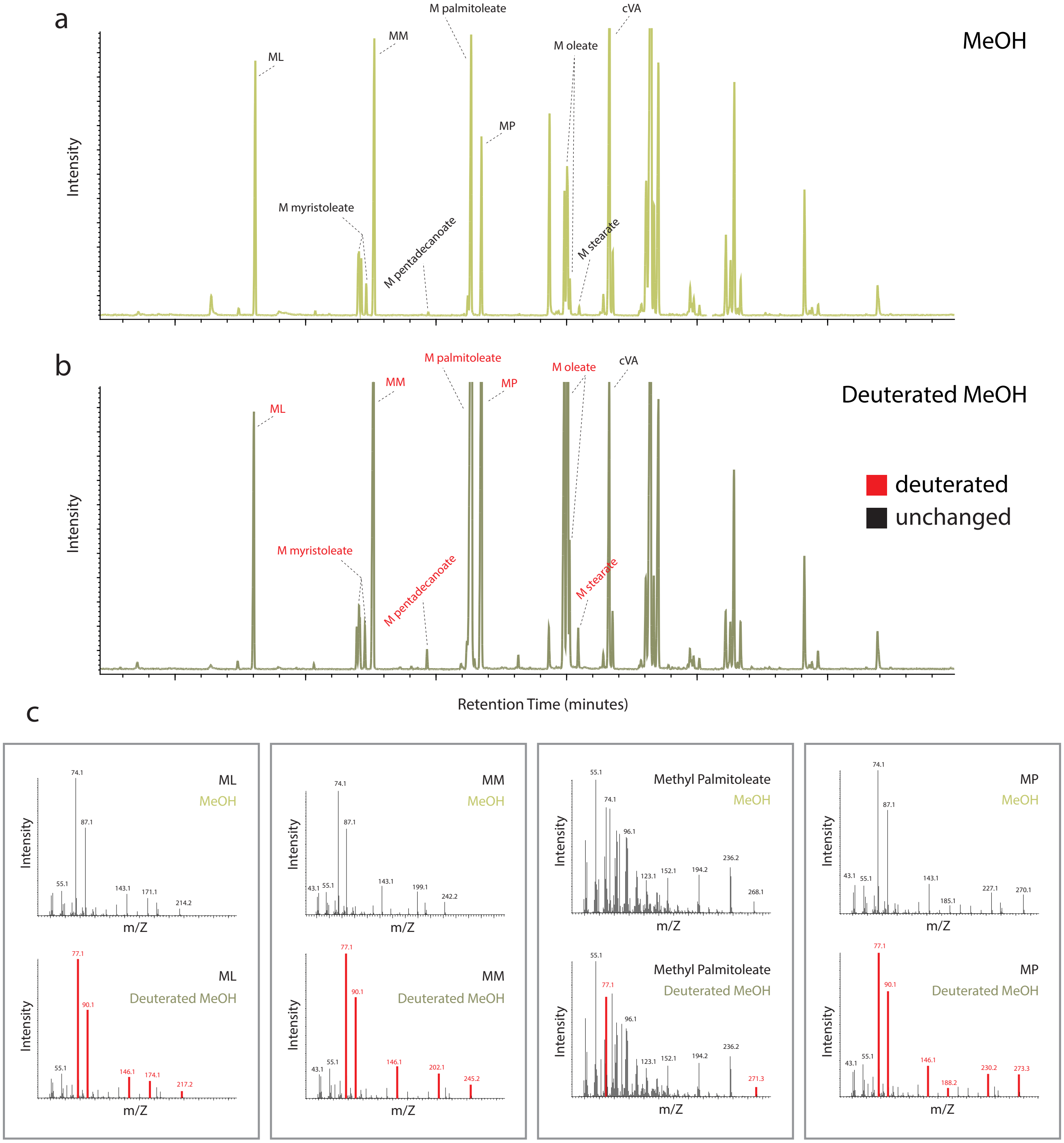

Figure S3. Use of deuterated methanol (d4) to assess pheromone increases.
Shown are examples of two GC-MS total ion chromatograms (TICs) of a single male D. melanogaster exposed to alcohol, with methanol at top (a), and deuterated methanol at bottom (b). Here we observe nearly identical TICs between these two treatments; however, several pheromone compounds have their molecular weight shifted in the case of the deuterated methanol treatment (bottom). This would include methyl laurate (ML), methyl myristate (MM), methyl palmitoleate (M palmitoleate), as well as methyl palmitate (MP). We did not observe any changes to other known pheromones, such as cVA, or cuticular hydrocarbons, such as 7-tricosane or 9-tricosane. Thus, this change in molecular weight following deuterated methanol exposure is limited to only those compounds that were increased following alcohol exposure. (c) Shown are the mass spectra of pheromones from methanol (top) and deuterated methanol exposed male Drosophila (bottom). In each case, we document a consistent shift in molecular weight for these fatty acid pheromones when the fly contacts deuterated alcohols, suggesting the pheromones are produced directly by the alcohol's donation of three deuterated hydrogen atoms. For example, from mass 74.1 to 77.1, or 87.1 to 90.1, which we observed in each example of pheromone increase.

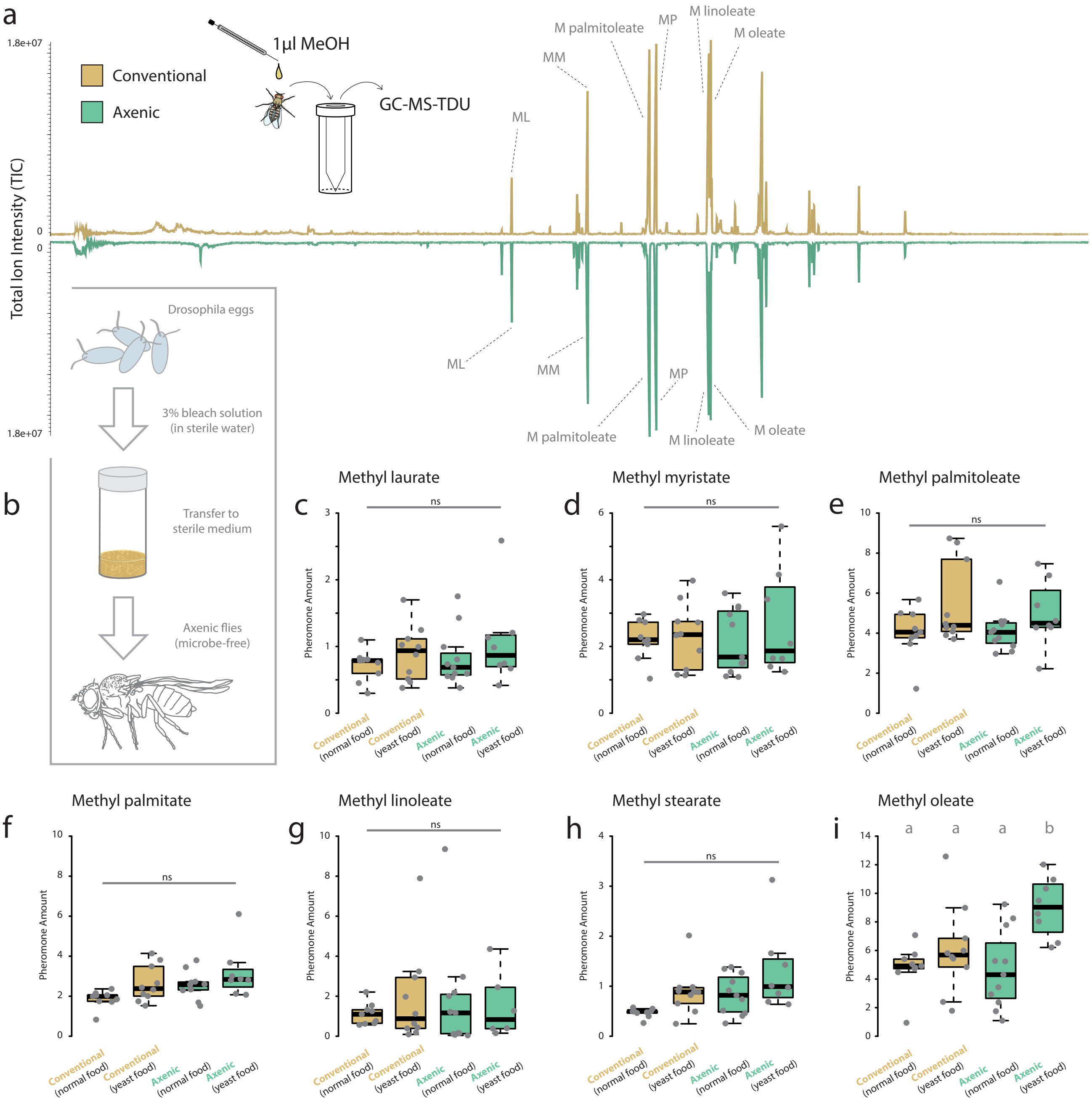

Figure S4. Pheromone comparisons between conventional and axenic Drosophila strains.
(a) TDU-GC-MS analyses of odor profile from single adult flies exposed to a droplet of methanol. Here we did not observe any significant differences in odor profiles between those flies grown with microbial symbionts (orange) and those without (green). (b) Diagram of methodology for axenic fly generation. Additional information available within the methods section. (c-i) Comparisons of average pheromone levels for flies grown with natural microbial symbionts (orange) and those without (green; axenic). We only note a single difference, within methyl oleate, and only for axenic flies grown on food with (non-living) yeast extract within their media (ANOVA with Bonferroni correction for selected pairs (tested were always axenic flies against conventional flies from the same food).

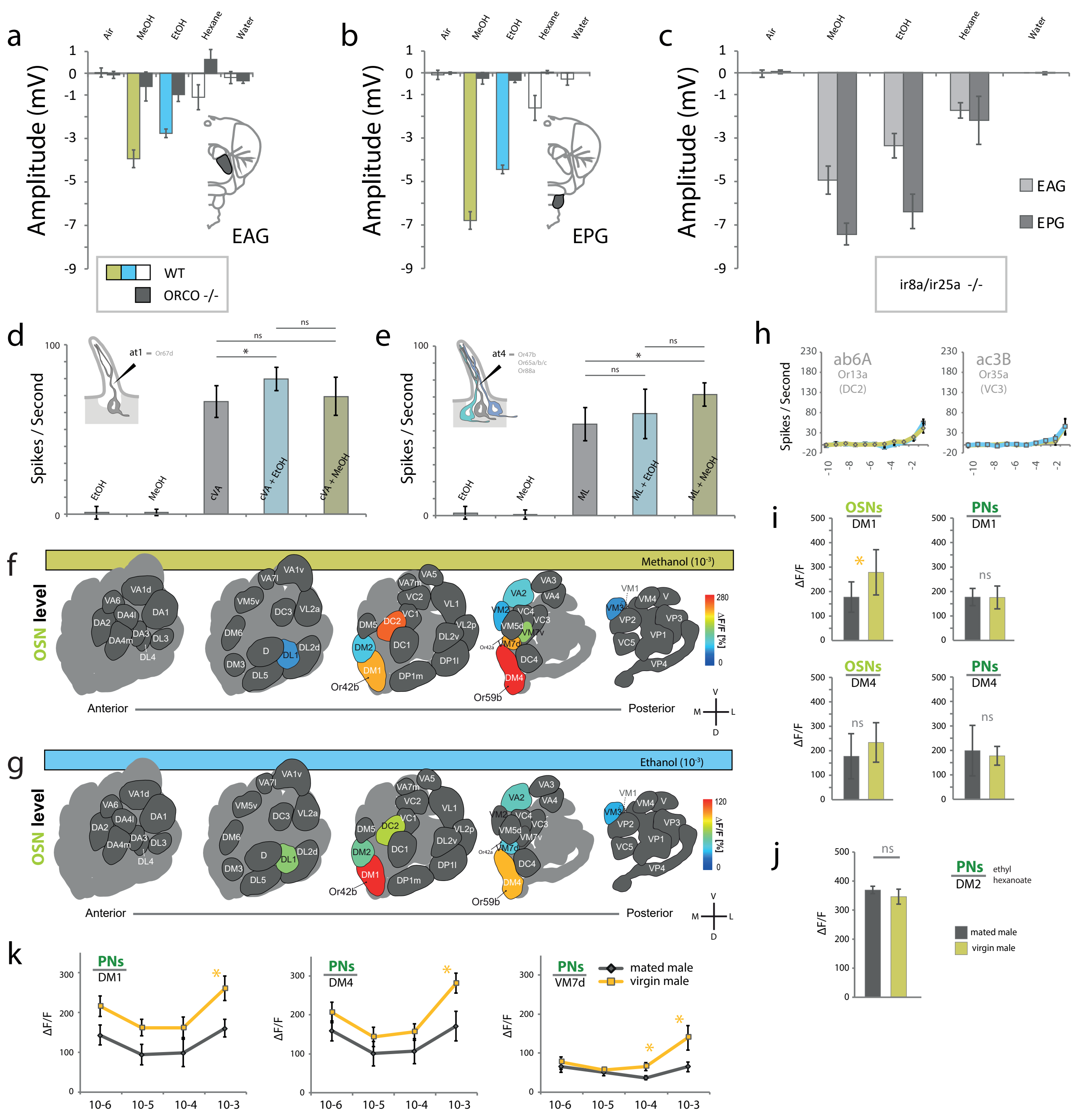

Figure S5. EAG, EPG, SSR and co-receptor mutants.
(a) Electroantennogram, showing that the antenna can strongly detect both alcohols, but not in Orco mutants. (b) Electropalpogram, showing that the palps of wildtype flies can detect both ethanol and methanol, but not in Orco mutants. (c) EAG and EPG recordings towards odorants using ir8a/ir25a double mutant for IR co-receptors. (d) SSR recordings from the at1 sensillum with alcohol exposure. Paired t-test (n=6) (e) SSR recordings from the at4 sensillum with alcohol exposure. Paired t-test (n=6) (f,g) Schematic antennal lobe (AL) atlas showing odor-induced calcium responses towards methanol and ethanol (10-3) in OSNs. Data collected from mated males. (h) Dose response for SSR. (i) Odor-induced calcium activity of OSNs and PNs of glomeruli DM1 and DM4 after stimulation with ethanol (10-3, n=5-6). (j) PN responses of DM2 to the non-alcohol odorant ethyl hexanoate (10-4, n=7-8) across physiological states, showing the specificity of changes for alcohol circuits. (k) Dose response curves of odor-evoked calcium responses for PNs to stimulation with methanol across mated and virgin males obtained with 2-photon functional imaging (n=6-9; two-tailed unpaired t-test).

Figure S6

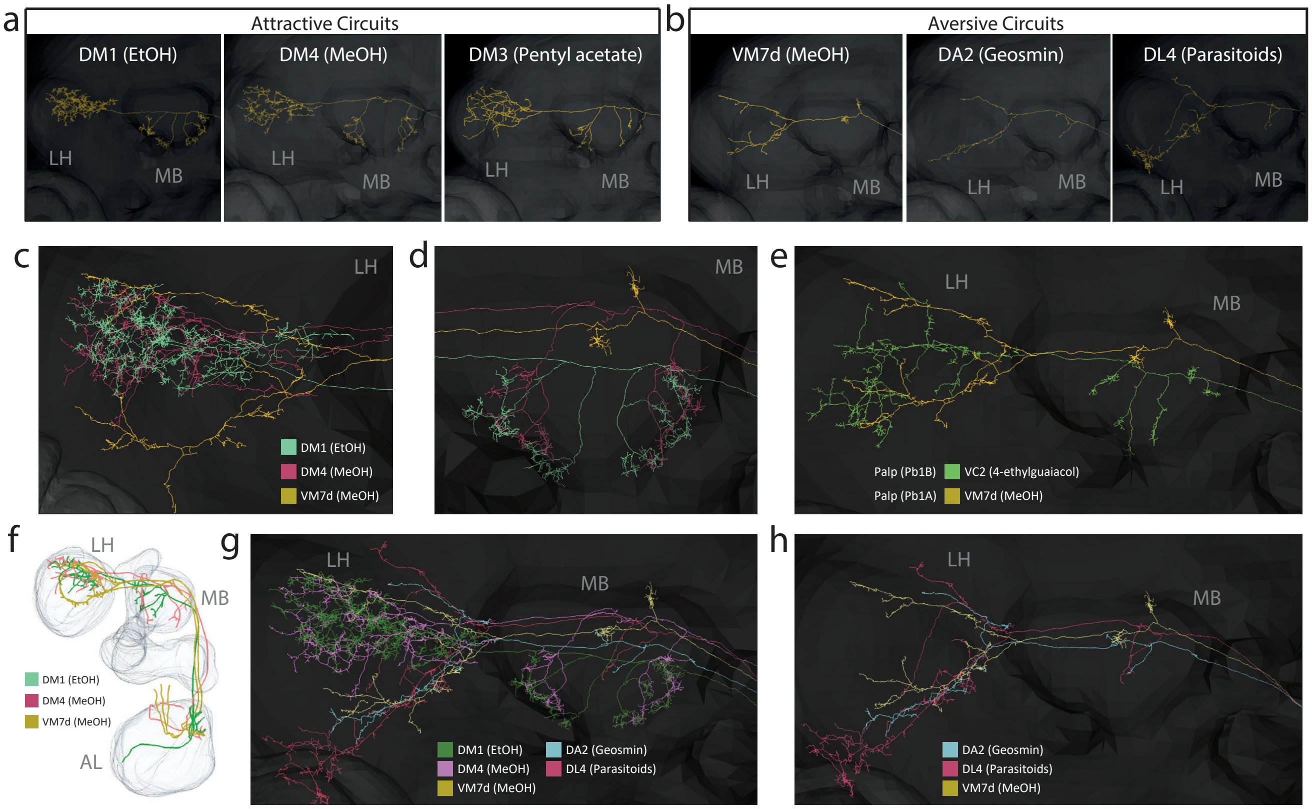

Figure S6. Neural reconstruction and circuitry related to alcohols.
Single projection neuron (PN) reconstructions from mushroom body (MB) into the lateral horn (LH). Reconstructions were obtained in our laboratory at MPICE during a 2017 PhD dissertation, and are not yet published 48, but were compared to and in agreement with other reference sets 49–51. Upper region of the LH is associated with innate attractive behavior, while the lower part is attributed to innate aversion. (a) Three single circuits associated with attractive behaviors. (b) Three single circuits associated with aversion. (c) Neural tracing of all three alcohol-related circuits within the LH, highlighting the difference between VM7d (yellow) and the two attractive circuits, DM1 and DM4. (d) Neural tracing of all three alcohol-related circuits within the MB, highlighting the difference between VM7d and the two attractive circuits, DM1 and DM4. (e) PN traces for both neurons extending from the pb1 sensillum showing that VM7d does not differ due to being from the palp rather than antenna. (f) PN reconstructions from the AL through the mushroom body (MB) and into the lateral horn (LH). Note that VM7d (aversion) has three PNs while the attractive circuits (DM1, DM4) have only one. (g) All examined alcohol-related circuits, overlaid with known aversive tracts (DA2, DL4), note also the high number of terminals in the MB for attraction but not aversive circuits. (h) VM7d circuit overlaid with known aversive tracts, showing high degree of overlap in LH as well as reduced MB branches.

a

Yeast Food*				
	Yeast	80	40	20
	Soy pepton	20	10	5
	Sucrose	30	15	7.5
	Yeast extract	20	10	5
	20% Glucose (mL)	300	150	75
	Agar	10	5	2.5
	qsp $H_2$0 (mL)	1000	500	250

Normal Food*				
	Corn flour	80	40	20
	Soy pepton	10	5	2.5
	Malt	80	40	20
	Yeast	18	9	4.5
	Sugar beet	22	11	5.5
	20% Glucose (mL)	100	50	25
	Agar	7	3.5	1.75
	Water (mL)	1000	500	250

*Add 40,20 or 10mL of Nipagin/EtOH solution (0.1g/mL) after autoclave

	Ingredients	1L	500mL	250mL
Apple-Juice*	Apple-Juice	250	125	62.5
	Agar	15	7.5	3.75
	ddH2O (mL)	750	375	187.5

Starvation*	Apple-Juice	250	125	62.5
	Agar	15	7.5	3.75
	20% Glucose (mL)	500	250	125
	ddH2O (mL)	250	125	62.5

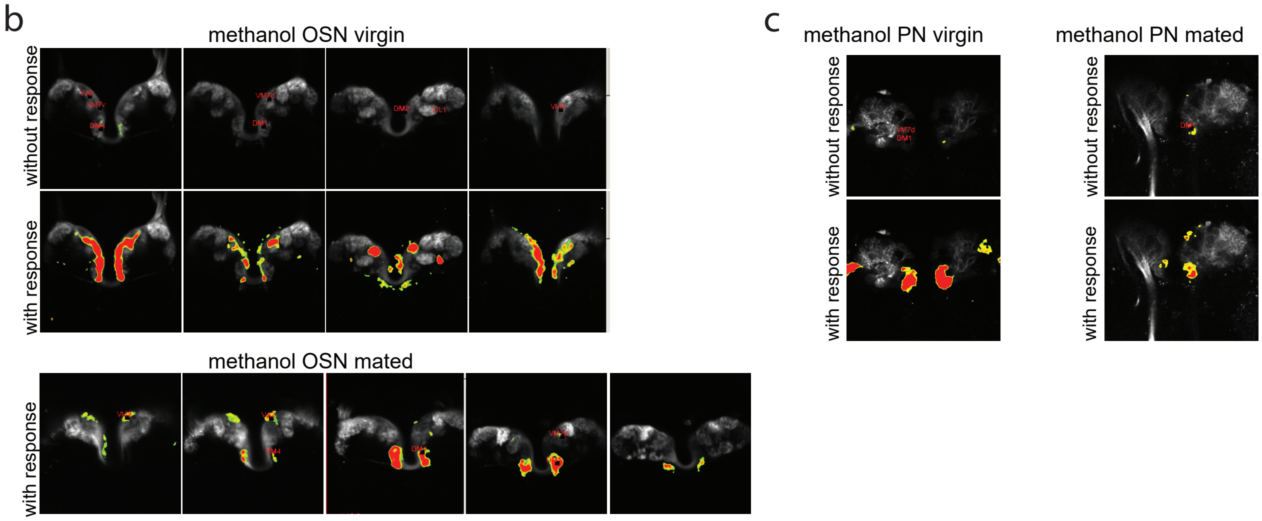

Figure S7. Diet (for axenic and gnotobiotic fly comparisons) and odor-evoked calcium responses
(a) Details are given for food types and media used for the generation and rearing of axenic and gnotobiotic flies (reference 66,67). Additional information available within the methods section of the text. (b-c) Representative odor-evoked calcium responses in PNs from mated and virgin males responding to methanol obtained through 2-photon imaging.

Supplementary Data S1. Currated Excel tables with raw data from each experiment. Available for download with the online version of this paper.
